# Trends and Stabilization up to 2022 in Overweight and Obesity in Switzerland, Comparison to France, UK, US and Australia

**DOI:** 10.3390/ijerph7020460

**Published:** 2010-02-11

**Authors:** Heinz Schneider, Eva S. Dietrich, Werner P. Venetz

**Affiliations:** 1 HealthEcon AG, CH-4001 Basel, Switzerland; E-Mail: edietrich@healthecon.com; 2 DataGen AG, CH-4310 Rheinfelden, Switzerland; E-Mail: werner.venetz@datagen.net

**Keywords:** prevalence, overweight, obesity, trend analysis, logistic regression

## Abstract

In Switzerland a rapid increase in the total overweight population (BMI ≥ 25) from 30.3% to 37.3% and in the obese segment (BMI ≥ 30) from 5.4% to 8.1% was observed between 1992 and 2007. The objective of this study is to produce a projection until 2022 for the development of adult overweight and obesity in Switzerland based on four National Health Surveys conducted between 1992 and 2007. Based on the projection, these prevalence rates may be expected to stabilize until 2022 at the 2007 level. These results were compared with future projections estimated for France, UK, US and Australia using the same model.

## 1. Introduction

Obesity is a chronic disease whose prevalence has been described as reaching “epidemic” proportions around the world [1,2]. Obesity is associated with a high risk of morbidity, mortality as well as reduced life expectancy [3]. The major health consequences of overweight and obesity are type 2 diabetes mellitus, all cancers except esophageal (female), pancreatic and prostate cancer, all cardiovascular diseases (except congestive heart failure), asthma, gallbladder disease, osteoarthritis and chronic back pain [4].

Particularly worrisome is the fact that among European school-age children (as an indicator of trends among older children and, eventually, adults in the decades to come), the prevalence rates of overweight are not only rising but also apparently accelerating [5]. It is most alarming that the current prevalence rate of childhood obesity in Europe and North America is more than 10 times higher than that of the 1970s. By 2010 it is estimated that 26 million children in the EU countries will be overweight, including 6.4 million who will be obese [6].

Switzerland is no exception among other European countries. Data from the fourth National Health Survey conducted by the Swiss Federal Institute of Statistics in 2007 showed that the prevalence of obesity in the general adult population, defined as body mass index (BMI) ≥ 30, had increased by 2.7% points to 8.1% since the first National Health Survey from 1992/93 [7]. For the entire adult Swiss population with BMI ≥ 25, a prevalence of 37.3% was observed in 2007 [8].

The goal of the present study is to address the question whether the observed alarming trend over the past 15 years may be expected to continue over the upcoming 15 years. Thus, a forecast until 2022 in the development of adult overweight and obesity in Switzerland was conducted through modeling based on the four National Health Surveys. For comparison, this model was also applied to other European countries (France, UK) as well as to the US and Australia.

## 2. Methods and Procedures

Our trend analyses were based on prevalence data from the Swiss National Health Surveys first instituted in 1992/3 and carried out by the Swiss Ministry of Statistics (Bundesamt für Statistik (BFS)) in 5 years intervals (see Table 1). The National Health Surveys of Switzerland are a series of phone surveys executed as part of an overall program of surveys commissioned by the Swiss Ministry of Health, *i.e.*, the *Bundesamt für Gesundheit* (BAG) designed to collect information on various health aspects of the adult Swiss population (age > 15 years) from a nationally representative sample by repeated use of the same questionnaire over time.

Target populations: In this study overweight and obesity populations were defined via BMI: overweight from 25–29.9 kg/m^2^, overweight and obesity as BMI ≥ 25, and obesity as BMI ≥ 30 kg/m^2^.

Data sources used for trend projections:

*Switzerland:* Previously published prevalence data on overweight and obesity of the Swiss population (age < 15 years) from the first three National Health Surveys (self-reported data) conducted in 1992/3, 1997 and 2002 were used in our trend analysis [7]. Furthermore, prevalence data from the fourth Health Surveys conducted in 2007 were available [8]. An additional data set refers to rates of overweight and obesity separated for young men and women of 15–24 years old.

*Other countries:* The prevalence data for overweight and obesity for the comparing countries—France, UK, USA (National Health Interview Survey, NIHS) and Australia—were obtained from the recently published OECD (Organization for Economic Co-operation and Development) Health Working Paper No. 45, which reported on the obesity epidemic in selected OECD countries [9]. All data provided in this report refer to rates of overweight and obesity separated for men and women aged 15–64 years old. With the exception of the UK (health examination survey), all data collections were based on National Health Interview Surveys [9].The former provides information on measured BMI, while the latter present self-reported figures.

Statistical analysis: The most natural model function for proportions is the logistic function as displayed in Equation (1)

(1) p=A/(1+EXP(B+C*Year)),

where p is the proportion of population and A, B and C are parameters to be determined. Equation (1) represents a sigmoid curve starting at 0 or 0% in earlier years and going towards A in the future. The maximum of A is 1, corresponding to 100%. Depending on the number of parameters used, we distinguish between the 2-parametric (with a fixed value for A) and the 3-parametric logistic function (where A is estimated together with parameters B and C). Using A as a fixed parameter we can estimate the parameters B and C by linearizing Equation (1) to

(2) log(A/p−1)=B+C*Year

and use the estimated intercept (B) and slope (C) as start values for the non-linear model of Equation (1). For the fixed value of A in the linear model, we used the 1.2-fold of the largest proportion p of the data (except for the obesity data of Australia: an A value of 0.3 was used). The assumption behind using an estimate of A below 1 is that never are all people either overweight or obese. These estimated parameters A, B and C are then used in the 3-parametric non-linear regression as start values for the curve fitting process. In case the 3-parametric logistic regression did not converge, we used the 2-parametric logistic regression model using A = 1. In order to assure that the data points were not in the asymptotic part of the S-shaped regression line, the descriptor variable *Year* was centralized before its use in the statistical model equation. Obvious outliers, most probably due to a biased population of the surveys, were eliminated as indicated in Table 2.

Equation (1) with the estimated parameters was then used to predict the proportion p for the year 2022. The uncertainty of this prediction is quite large. It becomes larger with increasing distance from the closest measured point, the smaller the number of actual measurements and the higher the spread around the fitted line. In addition, the predictions are more reliable if the measuring points are located in the upper part of the S-shaped curve. Curves and predictions for the intermediate BMI segment between 25 and 30 are calculated as the difference between the BMI ≥ 25 and BMI ≥ 30 curve values.

The results presented show the expected proportions of the populations of the various countries analyzed that will be overweight or obese in 2022. The obtained prevalence rates were plotted as non-linear curves. The parameters A, B and C of these curves are given in Table 2. SAS^®^ statistical software version 9.2 and R version 2.8.1 (R Development Core Team (2008)) were used.

## 3. Results and Discussion

### 3.1. Observed Prevalence of Overweight and Obesity in Switzerland from 1992 to 2007

**Figure 1.**
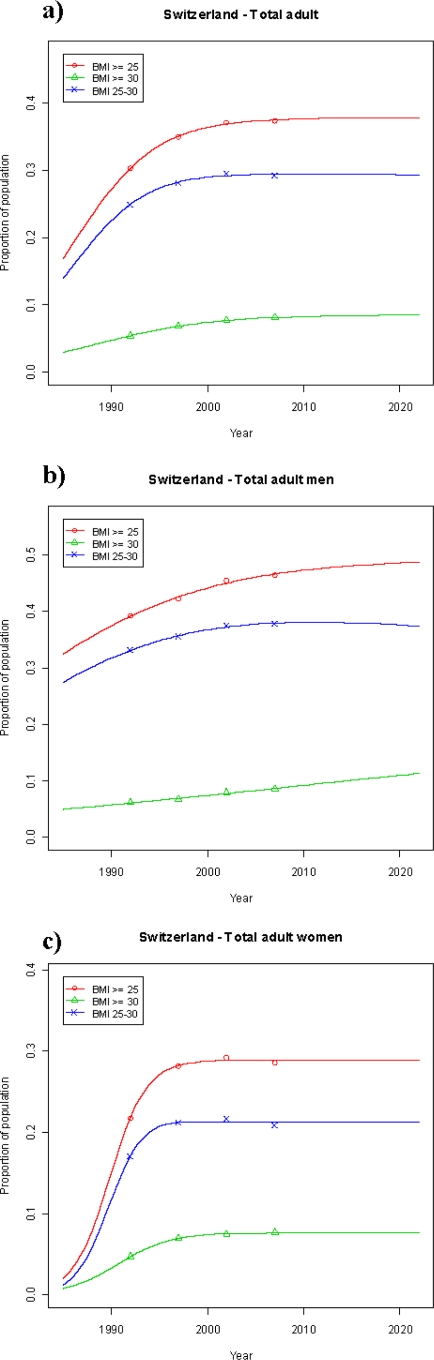
Prevalence of overweight (o), obesity (Δ) and overweight & obesity (x) among Swiss adults (age > 15): observed during 1992/93–2007 and projected (solid lines) up to 2022. The projected prevalence rates presented here are those based on our logistic regression estimation. a) Total adult, b) total adult men, c) total adult women.

*Total population:* All estimates are based upon prevalence data from the four National Health Surveys (telephone surveys) conducted by the Swiss Ministry of Statistics (Bundesamt für Statistik (BFS)) between 1992 and 2007 [7,8,10,11]. The development of overweight and obesity (BMI ≥ 25) over the period between 1992 and 2007 is shown in Table 1 and summarized in Figure 1, which clearly shows that the part of the adult population with surplus weight increased by 7% over these 15 years. This development is particularly obvious in the male population (increase by 7.2%), whereas it appears that a steady state may have been reached in the female population in this period (minimal increase of 0.4%).

*Age segment 15–24:* The actual development of the overweight prevalence over the past 15 years in the youngest segment of the adult Swiss population, *i.e.*, the age group between 15 and 24 years, is considered indicative for the trend in future development of the entire overweight segment. For this reason we analyzed this population segment separately.

As shown in Table 1, the population with BMI ≥ 25 in this age segment increased by 1.9% from 1992 to 2007. The increase in overweight individuals (BMI 25–29.9) amounted to 1% and the obese population (BMI ≥ 30) grew by 0.9%. The male part of the young adult population is predominantly responsible for this increase, whereas the female segment may have entered a steady state after the peak in 1997.

### 3.2. Projected Prevalence of Overweight and Obesity in Switzerland from 2007 to 2022

*Total population:* The above described data from the four available Swiss Health Surveys were used to model future trends for the development of obesity and overweight prevalence rates in the adult Swiss population until 2022. The expected proportions of adult Swiss population that are estimated to be overweight and obese by 2022 are shown in Figure 1.

The projection until 2022 yields an estimated minimal increase to 37.7% (from 37.3%) in the adult Swiss population with BMI ≥ 25, indicating a stabilization compared to the preceding 15 years period at a level below 40% (Figure 1a and Table 2a). The corresponding prevalence in 2022 in the population segment with BMI 25–29.9 is expected to be around 29.3%, a marginal increase by 0.1% from 2007. The projected small increase in prevalence from 8.1% in 2007 to 8.4% in 2022 in the obese population segment (BMI ≥ 30) underscores the likelihood that the increase in the obese Swiss population may come to a virtual halt in the upcoming two decades.

In the adult Swiss male population with BMI ≥ 25, the projected development shows a slow down in the anticipated increase to reach an approximate prevalence of 48.7% from 46.4% in 2007 (Figure 1b). The expected prevalence in 2022 in the male overweight segment (BMI 25–29.9) of around 37.4%, down from 37.8% in 2007, confirms the projected levelling out in the male overweight population.

A potential prevalence of 28.9% by 2022 can also be expected for the adult female Swiss population with BMI ≥ 25, compared to 28.6% in 2007, indicating a stable situation (Figure 1c, Table 2a). Similarly, the female overweight segment (BMI 25–29.9) is expected to be around 21.2% in 2022, virtually unchanged to 2007 (20.9%) and the prevalence in the obese female segment (BMI ≥ 30) is anticipated to remain at 7.7%.

*Age segment 15–24:* The projection of the assumed prevalence in 2022 anticipates an increase to 12.8% (from 11.7% in 2007) in the young adult population with BMI ≥ 25 (Table 2a). The prevalence is expected to decrease to 8.5% by 2022 from 9.9% in 2007 in the overweight segment (BMI 25–29.9), whereas the prevalence in the obese segment (BMI ≥ 30) may eventually increase to 4.3% from 1.8% in 2007 (Table 2b).

### 3.3. Projected Prevalence of Overweight and Obesity from 2007 to 2022 in Other Countries

The use of non-linear logistic regressions described above provides a more accurate picture of overweight and obesity trends over time within a country than linear trend estimation. This approach may also be more useful to compare future trends across countries, provided that sufficient data from repeated cross-sectional national surveys over longer time periods are available.

Recently published data available from repeated cross-sectional national surveys from France, UK, US (NHIS) and Australia [9] allowed the projection of the development of overweight and obesity prevalence until 2022 using our logistic curve fitting model. As shown in Figure 3a, the development of overweight prevalence (BMI ≥ 25) of the adult population in the two central European countries—Switzerland and France—over the past years was comparable and is expected to reach around 40% until 2022 in both countries (Table 2a). An overlapping development in overweight prevalence has been observed between 1990 and 2005 in the UK and the US, reaching a significantly higher level (around 60%) than in central Europe. Future projections anticipate a levelling out above the 60% mark by 2022. In Australia, an almost linear increase in overweight prevalence was observed from 1989 until 2004 and the trend analysis foresees a continued increase to a level of about 65% in 2022.

In Figure 3b the corresponding developments for the prevalence of obesity in the five countries are shown. Switzerland and France show a comparable development since 1990 until present, reaching obesity prevalence rates around 8%–9%. In the case of Switzerland, trend analysis indicates stabilization over the next 15 years whereas a continued increase may be expected to occur in France up to a prevalence of about 17% in 2022. For the UK and the US, the projections predict a continued increase in obesity prevalence in both countries eventually reaching a level around 30% (Table 2b). In Australia, a continued increase up to approximately 25% may be expected until 2022.

## 4. Discussion

As reported by the International Association for the Study of Obesity (IASO), the prevalence of obesity as defined by BMI ≥ 30 has surpassed the 20% population mark in many European countries [12]. As a consequence, overweight and obesity are frequently making media headlines and are amongst the highest priority public health issues in the world [13]. To make matters worse, current evidence suggests that the prevalence of overweight and obesity is likely to remain on the rise in the US [14], developing countries [15] as well as Europe [16]. Taking this worldwide development in obesity into account, one central question arises: When will this global trend of increasing overweight and obesity come to an end?

The present study aimed at providing a possible answer to this question with respect to the overweight and obesity situation in Switzerland. Between 1992 and 2007 a rapid and significant increase in the prevalence of overweight and obesity (BMI ≥ 25) in the adult Swiss population (age > 15) by 7% to a total of 37.3% was observed, consisting of a predominant increase in the proportion of overweight individuals (BMI 25–30) by 4.3% and a concomitant increase of the obese population by 2.7%.

The projection until 2022 based on the above prevalence data by a logistic regression model offers an estimated minimal increase to 37.7% in the prevalence of the overweight and obese segment of the Swiss population (BMI ≥ 25), indicating stabilization at a plateau below 40%. This interpretation is supported by trend analysis of the youngest segment (age 15–24) of the adult overweight and obese population with BMI ≥ 25, as the projection of the assumed prevalence in 2022 shows only a small increase by 1.1%. Regarding the expected obesity prevalence in 2022, the projected value is 8.4% in the entire Swiss population, a slight increase by 0.3% compared to 2007, again indicating stabilization at a level below 10%. Although the projected value for 2022 in the youngest population segment (age 15–24) increases to 4.3% from 1.8% in 2007, it does not necessarily contradict the trend towards stabilization expected in the entire Swiss population. It has to be kept in mind that the projection in the young obese population segment is based upon a small number of individuals with a corresponding high degree of uncertainty.

**Figure 2.**
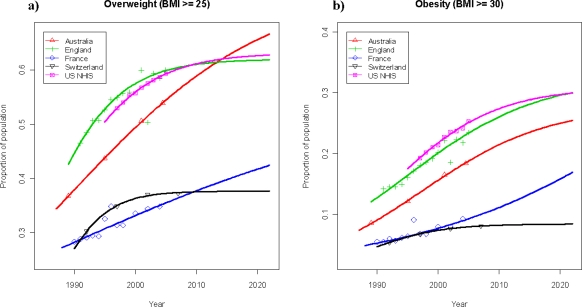
Prevalence of overweight (BMI ≥ 25) and obesity (BMI ≥ 30) among the adult populations (>15) of the US (NHIS), UK, Australia, France and Switzerland: observed from 1989–2007 and projected (solid lines) up to 2022. The projected prevalence rates presented here are those based on our logistic regression estimation.

Our prediction for the expected development of overweight and obesity in Switzerland is not as grim as a recent projection for the US, which forecasts that, by 2048, all American adults would become overweight or obese, while black women will reach this state by 2034 [17]. In the context of this gloomy projection for the US population, it has to be mentioned that for this US projections a linear regression model was used to extrapolate future prevalence rates. Obviously, a simple linear model for proportional data is only appropriate in the approximately linear region of the S-shaped curve and does not allow projections in the asymptotic part of the curve. Using a logistic regression model approach with a 3-parameter logistic function, however, not only accounts for the slowdown of the rates at both ends of the curve but also allows the overweight population to reach a limit below 100% (parameter A < 1), while attaining a close match to the data already available. Thus, using the US-NHIS data in our model, the US overweight prevalence rate is projected to level out until 2022 around 63% from 59.3% in 2005. The same levelling of the US obesity trend was found in projections produced in 2006 by the Centers of Disease Control and Prevention [18]. Furthermore, our findings were recently confirmed in the context of a study projecting the future diabetes population in the US and describing that the overall distribution in the non-diabetic population remains fairly stable with ~65% being overweight or obese [19]. Interestingly, the curve obtained from the UK prevalence data between 1991 and 2005 is virtually matching the US prevalence curve (Figure 2a). A similar overlap of obesity prevalence curves, both observed and projected, between the US and the UK results from our model approach indicates that the situation in the past and the future appears to be comparable between these two countries (Figure 2b). A similar development in overweight and obesity prevalence has also been observed in 1990–2007 between Switzerland and France, but at lower levels than in the US and the UK. The projections predict a levelling out for overweight until 2022 in both countries eventually reaching a level between 38% and 42%, compared to approximately 63% in the US and the UK. While the obesity prevalence in France may increase to about 17%, the situation in Switzerland is expected to remain stable (8.4%), less than a third of the obesity prevalence projected for the US and the UK. The most rapid increase in overweight prevalence over time has been experienced in Australia (Figure 2a). Trend analysis results in the highest expected overweight prevalence for all five countries evaluated to about 65% in 2022. In support of our findings, similar projected trends regarding France, US, UK and Australia have been described in the recently published OECD working paper [9].

An interesting finding of the present study refers to the obvious differences in overweight and obesity prevalence rates observed between the evaluated countries, particularly between Switzerland and France on one side and the US, UK and Australia on the other. Unfortunately, there is no simple explanation for the observed, obvious differences between these industrialized countries. It may be speculated that differences in the obesogenic environments (aspects of physical, social and economic environments that favour obesity development) exist between the US, UK and Australia and the two central European countries that could be responsible for the observed differences in overweight and obesity prevalence rates developed over time. It has been argued that obesity represents a life-style situation heavily influenced by genetic heritage [20]. Genetics alone, however, cannot account for the increase in overweight and obesity encountered in many countries over the past 2–3 decades [9]. Although socioeconomic difference in obesity prevalence rates are well documented, in countries where the largest socioeconomic disparities exist, overweight and obesity rates are not necessarily the highest [9]. Overweight and obesity may also be viewed as environmental (social) phenomena promoting physical inactivity and excessive food consumption [21]. Research showed that the spread of obesity in social networks (family, peer groups) appears to be another important factor in the global obesity epidemic [22]. In summary, it appears that obesogenic environments that promote physical inactivity and excess (fat) food consumption may have encouraged individuals, particularly when socially and culturally vulnerable, to chose/accept less healthy life styles, and those with a genetic predisposition may have ended up becoming overweight and obese [9]. Differences in overweight and obesity among individuals with different levels of education are remarkably consistent across countries. In most countries a gradient is observed: the lower the education attainment, the higher the likelihood of being obese or overweight [9]. Switzerland is no exception as the obesity prevalence also decreases with higher educational level in both genders [22]. The fact that obesity and overweight are higher among men and women with lower educational attainment does not explain the observed differences in overweight and obesity prevalence rates developed over time between the US, UK and Australia on one hand, and France and Switzerland on the other hand.

Our study has several limitations. The major limitation is the fact that the model used for our projections is based on a number of assumptions. We assume that it is highly unlikely that the entire population of a specific country may turn overweight or obese. Thus, we calculated an upper limit for both cases based on presently available prevalence data, assuming that the upper asymptotic part of the S-shaped curve comes to lie below 100% (parameter A < 1) as a close reflection of the real situation. Future observations may disprove these assumptions. Furthermore, the estimation of the prevalence rates may not be absolutely comparable in the five countries evaluated since the methods of estimation differed among the countries (surveys *vs*. actual measurements) [24,25]. For example, from the two different surveys undertaken in the US, the National Health and Nutrition Examination Survey (NHANES) and the National Health Interview Survey (NHIS), the former provides information on measured BMI, while the latter presents self-reported figures. Self-reported rates from the NHIS appear to under-estimate obesity compared to actual rates reported in NHANES, but the time trends are the same [9].

In conclusion, after a rapid increase in the total overweight population (BMI ≥ 25) as well as in the obese population segment (BMI ≥ 30) in Switzerland over the past 15 years, the prevalence rates may be expected to basically stabilize between 2007 and 2022 at about the 2007 level. In all four countries (France, US, UK, Australia) with nationally representative data collections over the past 1–2 decades evaluated for comparative reasons, a rapid increase in the prevalence of overweight and obesity occurred during the last 10–20 years. While the development in France, compared to Switzerland, showed a similar increase in overweight and obesity over a comparable time period, the other countries (US, UK, Australia) reached considerably higher proportions in the overweight and obese segments of the adult population. The projection based on the presently available overweight and obesity prevalence data using logistic regression analysis yielded a levelling off with respect to the expected overweight prevalence rates in Switzerland, France, the US and the UK until 2022, whereas a continuous increase in overweight is expected to occur in Australia. Based on this projection it appears that the unprecedented increase in overweight prevalence in some industrialized countries may come to an end in the next decade. With the exception of Switzerland, obesity prevalence rates are still expected to rise until 2022 albeit at a slower pace. Countries with a historically “low” obesity level, such as Switzerland and France, may not catch up to the countries with much higher obesity prevalence rates (US, UK, Australia) within the upcoming decades for reasons unknown at the present time.

## Figures and Tables

**Table 1.** Results of the Swiss National Health Surveys from 1992 to 2007 [7,8,11,12].

**Nat. survey**	**Total adult population (age > 15)**			**Overweight & obesity BMI ≥ 25**			**Overweight BMI 25 – 29.9**			**Obesity BMI ≥ 30**		
	**Female**	**male**	**total**	**female**	**male**	**total**	**female**	**male**	**total**	**female**	**male**	**total**
	total number	total number	total number	**%**	**%**	**%**	**%**	**%**	**%**	**%**	**%**	**%**
1992/93	2 947 789	2 735 471	5 683 260	21.8	39.2	**30.3**	17.1	33.1	**24.9**	4.7	6.1	**5.4**
1997	3 052 211	2 827 975	5 880 186	28.2	42.2	**34.9**	21.2	35.5	**28.1**	7.0	6.7	**6.8**
2002	3 108 453	2 909 185	6 017 638	29.3	45.4	**37.1**	21.8	37.5	**29.4**	7.5	7.9	**7.7**
2007	3 164 763	3 021 948	6 186 711	28.6	46.4	**37.3**	20.9	37.8	**29.2**	7.7	8.6	**8.1**
	**Total population (age 15–24)**			**Overweight & obesity BMI ≥ 25**			**Overweight BMI 25 – 29.9**			**Obesity BMI ≥ 30**		
	**female**	**male**	**total**	**female**	**male**	**total**	**female**	**male**	**total**	**female**	**male**	**total**
	total number	total number	total number	**%**	**%**	**%**	**%**	**%**	**%**	**%**	**%**	**%**
1992/93	423 107	432 646	855 752	6.8	12.9	**9.8**	6.1	11.8	**8.9**	0.7	1.1	**0.9**
1997	400 874	406 866	807 739	9.4	11.9	**10.5**	8.4	10.8	**9.6**	0.8	1.1	**0.9**
2002	414 663	430 144	844 807	7.9	14.3	**11.2**	5.4	12.7	**9.1**	2.5	1.6	**2.1**
2007	458 298	486 648	944 947	7.7	15.4	**11.7**	6.0	13.6	**9.9**	1.7	1.8	**1.8**

**Table 2a.** Logistic regression results for overweight (BMI ≥ 25): Model parameters A, B and C of Equation 1. If parameter A = 1 then the 2-parameter model was used. n = number of survey years.

**Country**	**Population**	**Age (y)**	**Outlier excluded**	**n**	**A**	**B**	**C**	**Pred. 2022**
Australia	Total	>15		4	0.7535	125.1	−0.0629	66.6%
	Men	>15		4	0.9926	102.0	−0.0512	80.5%
	Women	>15		4	0.5769	151.0	−0.0759	53.5%

France	Total	>15	1996,0.349	11	0.5494	72.3	−0.0364	42.4%
	Men	>15	1996,0.426	11	0.4775	155.3	−0.0785	46.2%
	Women	>15	1996,0.283	11	1	45.1	−0.0221	36.5%

Switzerland	Total	>15		4	0.3774	459.9	−0.2316	37.7%
	Men	>15		4	0.4936	195.3	−0.0987	48.7%
	Women	>15		4	0.2890	1050.6	−0.5280	28.9%

Switzerland	Total	15–24		4	0.1385	108.1	−0.0547	12.8%
	Men	15–24		4	1	36.4	−0.0173	18.8%
	Women	15–24		4	1	9.7	−0.0036	8.5%

UK	Total	>15	2002,0.503	13	0.6197	317.1	−0.1598	61.8%
	Men	>15	2002,0.532	14	0.6610	385.6	−0.1943	66.1%
	Women	>15	2002,0.479	14	0.5983	249.7	−0.1259	59.3%

US NHIS	Total	>15		9	0.6321	264.4	−0.1332	62.8%
	Men	>15		9	0.7023	336.6	−0.1696	70.1%
	Women	>15		9	0.5968	177.4	−0.0894	57.7%

**Table 2b.** Logistic regression results for obesity (BMI ≥ 30): Model parameters A, B and C of Equation 1. If parameter A = 1 then the 2-parameter model was used.

**Country**	**Population**	**Age (y)**	**Outlier excluded**	**n**	**A**	**B**	**C**	**Pred. 2022**
Australia	Total	>15		4	0.2778	193.8	−0.0970	25.4%
	Men	>15		4	0.6587	138.8	−0.0688	38.4%
	Women	>15		4	0.2063	254.5	−0.1278	20.2%

France	Total	>15	1996,0.091	11	1	83.3	−0.0404	16.9%
	Men	>15	1996,0.089	11	1	83.0	−0.0403	16.9%
	Women	>15	1996,0.093	11	1	83.8	−0.0407	17.0%

Switzerland	Total	>15		4	0.0847	340.2	−0.1711	8.4%
	Men	>15		4	0.1800	81.1	−0.0404	11.3%
	Women	>15		4	0.0768	737.0	−0.3702	7.7%

Switzerland	Total	15–24		4	1	108.5	−0.0521	4.3%
	Men	15–24		4	1	81.3	−0.0385	3.2%
	Women	15–24		4	0.0212	707.2	−0.3543	2.1%

UK	Total	>15	2002,0.186	14	0.3245	183.6	−0.0920	30.0%
	Men	>15		15	0.2780	228.3	−0.1145	26.8%
	Women	>15	2002,0.192	14	0.4630	119.1	−0.0594	34.7%

US NHIS	Total	>15		9	0.3082	238.8	−0.1198	29.9%
	Men	>15		9	0.2910	293.7	−0.1474	28.7%
	Women	>15		9	0.3253	202.1	−0.1014	30.9%
